# Supplementary material for: Functions of Hemp-Induced Exosomes against Periodontal Deterioration Caused by Fine Dust
Source: Int J Mol Sci. 2024 Sep 25;25(19):10331. doi: 10.3390/ijms251910331 (PMC11477052; doi:10.3390/ijms251910331)
Supplement: Supplementary file 1 [file ijms-25-10331-s001.zip › File S1.pdf]

Evaluation of CBDVA from cheongsam matured stem extract, hemp in Andong city, Korea

- LC-MS/MS

Instrument: Agilent Technologies 6410 Triple Quad (LC-MS/MS)
Column: Unisol C18 (3.0 mm × 150 mm, 3.0 ㎛)
Solvent: A; 0.1% Formic acid in Water, B; 0.1% Formic acid in Acetonitrile
Flow rate: 0.4 mL/min
Injection vol.: 5 mL
Gradient :

	time(min.)	B%
1	0	70
2	12	100
3	15	100

- MS condition

Ionization Mode	-ESI, scan mode
Gas temp.	350 ℃
Capillary volt.	4000 V
Nebulizer	40 psig
Fragmentor	135 V

- CBDVA : CAS Number 31932-13-5

Results

Standard (CBDVA)

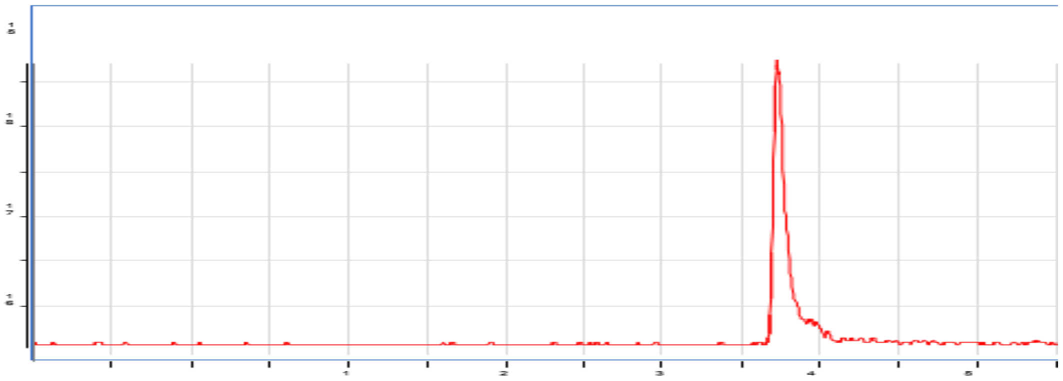

MSE (CBDVA)

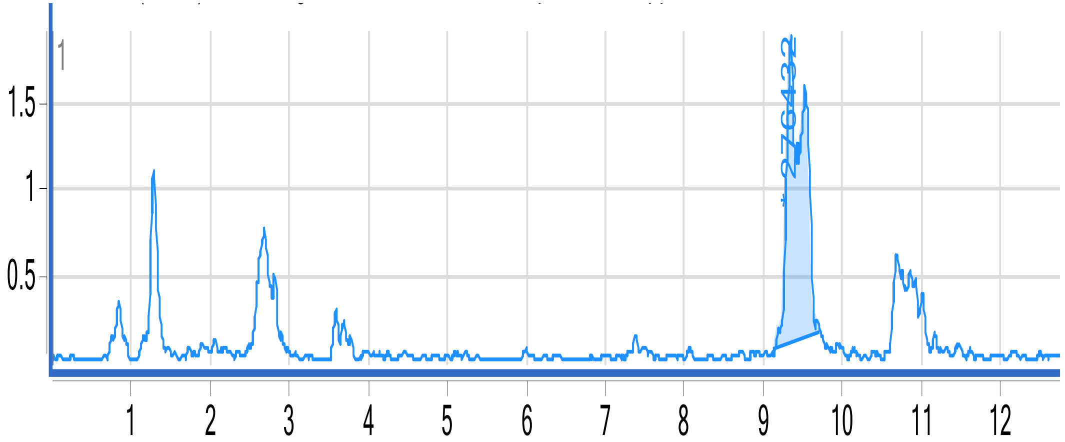

MSE ; matured hemp stem extract

Calibration curve

Compounds	Regression equation	Linear range	Correlation coefficient
		(mg/mL)	(R^2)
CBDVA	y = 622,021.0959x - 5,764.0411	0.5-5	1.0000

Results

Sample		
	Area	Sample 1mg
MSE	276,432	0.009 µg

Flavonoids and α-tocopherol in MSE

Flavonoids : Plant Flavonoids Colorimetric Assay Kit, Elabscience
α-tocopherol : Alpha-Tocopherol ELISA Kit, abbexa

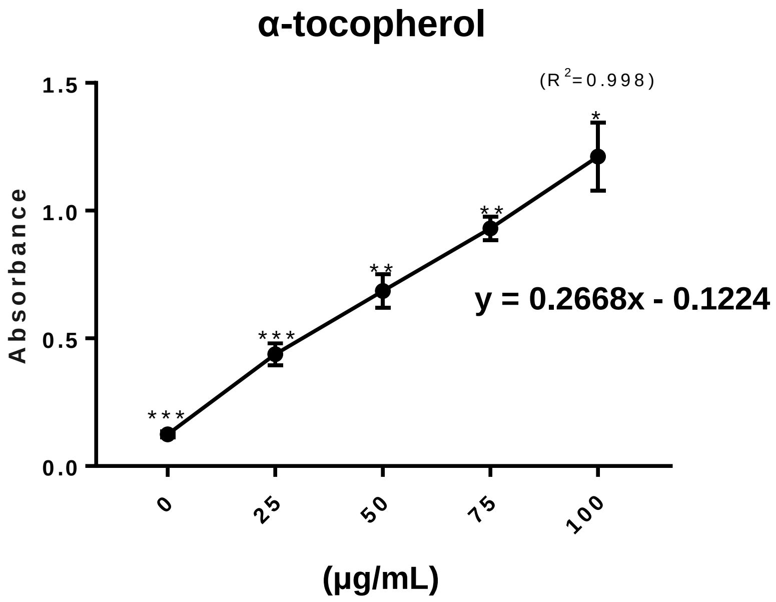

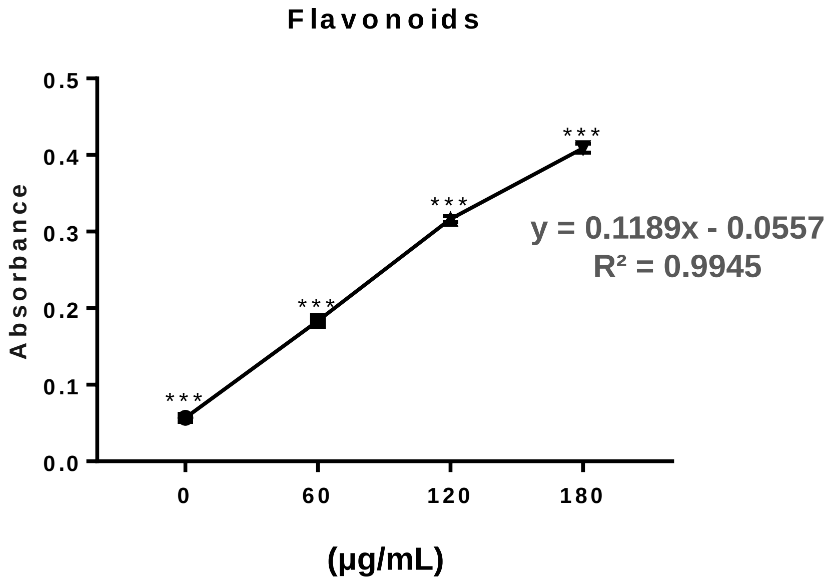

Result : 0.624 μg/ MSE 1mL

Result : 12.37 μg/ MSE 1mL

(*: p < 0.05, **: p < 0.01, ***: p < 0.001)
